# Preadamite Races of Men

**Published:** 1881-01

**Authors:** Harvey L. Byrd

**Affiliations:** Baltimore, Md.; Formerly Professor of Materia Medica and Therapeutics, and Principles and Practice of Medicine, and late Professor of Obstetrics, and Diseases of Women and Children, etc., etc.; 225 North Gilmor Street


### Independent Practitioner.

VOL. II.——*January 1881.*——NO. 1.

ARTICLE I.

### PREADAMITE RACES OF MEN.

BY HARVEY L. BYRD, A. M., M. D., BALTIMORE, MD.

Formerly Professor of Materia Medica and Therapeutics, and Principles and Practice of Medicine, and late Professor of Obstetrics, and Diseases of Women and Children, etc., etc.

Whether the races of men are contemplated from a biblical, archæological, ethnological or medical standpoint, the subject presents the deepest interest, and one naturally realizes, at a very early period of the investigation, that he is personally concerned in its elucidation and result. Much light has been thrown upon the origin and distribution of the races of men, (as they exist at present,) during the past half century, and the last decade has probably been productive of more satisfactory results in regard to it than were developed within the entire historic period by which it was preceded. Perfect harmony is found to exist between the Mosaic statements and the ethnological idea of a plurality of races existing at the time of Adam, by those who carefully study the subject. The investigation of race peculiarities is certainly of the highest importance to the physician, as well as the ethnologist, and as such the pages of a medical journal are particulay well suited for discussing them. A proper appreciation of the text in Genesis will show conclusively that Cain found his wife in the land of Nod, among a people who were to be his future countrymen, and against whose violence and wrath the "Mark in his forehead," (probably the sign of the cross) was to afford him protection. And that "the sons of God," sons of Adam, obtained their wives from among the "*fair* daughters of men," is explicitly declared in the sacred text.—(Gen. 6, 1 and 2.)

These latter inter-marriages were *necessary* in order to the propagation and perpetuation of the Adamite or Jewish race. This I have endeavored to show in several published communications. First in a brief article in the *Oglethorpe Medical and Surgical Journal*, Vol. III, p. 143 (1860), under the caption, "*Did all the Races of Men Originate from a Single Pair?*" Ten years later, and after careful study and investigation of the subject, much of the original article was re-produced in the *Baltimore Medical Journal*, Vol. I, p. 426 (1870), under the caption, "*Are All the Races of Men Descended from a Single Pair?*" In the same *Journal*, Vol. I, p. 529, I have another article, "*Anatomical and Physiological Differences between the Caucasian and African Races.* And in 1875 I published several articles in the *Baltimorean*, a literary weekly newspaper of this city, in which the subject of the plural or diverse origin of the human races was contended for, and effort made to reconcile such a theory with the teaching of the Bible, etc., etc.* Further study, and increased facilities for investigating the subject, have strengthened my belief in the *diversity* of origin, and *great* antiquity of the negro, mongoloid and white types of mankind. *All* of them antidating the creation of Adam, the head and parent of the Jewish race, doubtless by many centuries. My opportunities for study and investigation have tended to establish and confirm the *immutability* of Nature's laws; and so far as I have been able to correctly interpret those *now* operating upon the higher orders of animal life, including the various types of mankind, I cannot perceive that there could have been development *out of* a *lower* into a *higher* grade, in any, much less in numerous instances, as has been contended for in the human, or in any of the other well-defined types of any genus of the animal creation! On the converse, there has been a creation, or development *from*, and not a growing *out of* a lower to a higher order of animals of the same, or similar genus, or family.

The Intelligence that originated the law under which the *protoplasm* came into being, or that gave activity to the *cell*, was quite sufficient for the creation and arrangement of *all* the other laws which we know are *necessary now* to the perpetuation and preservation of the various races of men! Entertaining this view of the Omnipotent Author of *all* the laws now governing the organic and inorganic world, I do not find it necessary to enter the hypothetical, or the domain of the

*This position was sustained by my friend Dr. G. Halsted Boyland, and others.

unreal, in order to account for the radical differences and distinctions which now so conspicuously mark the races of mankind.

The three primordial types, already named, were, at least, separate and distinct creations; and no amount of sophistry, ingenious reasoning, nor special pleading can ever be able to change the decree of nature in making them what they are and were created! Take, for example, the Caucasian and Negro races, under cognizance of the senses, and with the scalpel and microscope as aids in the demonstration, and the greatest differences and distinctions will be found to exist, *as great* at least, if not greater even, than those existing between the horse and the ass; or the eagle and the owl! Both are separate and distinct creations, and as such the relationship is not closer between the white man and the negro, than that between the horse and the ass, or the eagle and the owl. In fact, when the same means are used in instituting comparison between the negro and chimpanze, he will be found more *nearly* allied to the latter than he is even to the Mongoloid races. *This is the teaching of science!* and not conjecture! *These are facts*, upon which the unprejudiced and thoughtful mind can rest in confidence and security. Were these differences and distinctions always such? We answer, they have undergone *no change* for fifty centuries, hence we are willing to assert that they are *not* more marked and obvious now than they were at the time of their original creation! There has, therefore, been *no transmutation* of the three great primordial types of mankind; the conjectures and hypothetical absurdities of animal evolution to the contrary notwithstanding. Accident, chance, contingency and verisimilitude are the offspring of human credulity, prejudice or ignorance; and in an intellect capable of relying upon the knowable and the known, cannot be supposed to have any weight either in the solution of problems or the determination of unvarying results! That there were not *one*, nor *two*, but probably several, if not numerous, foci of creation, can, or ought to be easily accepted by an unprejudiced mind! Though the genus homo is cosmic to a great extent, and some of the races capable of existing in lands and climates differing widely from their autochthonic homes, they live and flourish best on their native soil and in their own or similar climes. One has but to cast an eye over a map of the world and observe the distribution of the fauna, and flora, in their wise and beautiful diversity throughout the climates and surface of the earth, and in the seas, to be convinced at once that Infinite Wisdom formed them all, upon a plan best suited to their wants and necessities. A plan that cannot err, nor ever be radically changed. Everywhere similar animals and plants are found inhabiting similar latitudes and climes; identical forms are, however, rarely seen in different continents, even of the same latitude; but all are adapted and suited to the regions for which they were designed by the creative Will. The *Ornithorhynchus* and the *Cervus Tarandus* have ever inhabited the antipodal climates in which their essentially diverse organisms were created, and the food upon which they live is a like native to the lands of their creation, and either would perish soon should their homes be diametrically changed. The Apteryx, the Sloth and the Gopher, afford additional and striking evidence of autochthonal origin. Each has undoubtedly continued to inhabit the region in which it was originally created! The first, a bird which can neither swim nor fly, is found no where else on the surface of the globe, except in New Zealand; the second, an animal of most tardy movement, primordially inhabiting trees in tropical South America, and scarcely capable of locomotion on the ground at all; and the last, a species of terrapin, that cannot swim, is found exclusively on dry land and burrows in the sand along the Atlantic and Gulf Coast, from the Savannah to the Mississippi Rivers, living on vegetable matter in the summer, and hibernating in the sand during the cold season. These animals were all adapted by the Creator to those portions of the earth's surface upon which they exist to-day, and the boundaries of the territories they inhabit are as natural and as distinct as those of the flora by which they are now, and have ever been surrounded since the period of their original formation. Other instances could be cited to show that there were not one, nor two, but doubtless many foci of creation of the fauna and the flora of the earth. All experience and the revelations of history, as well as the forms and habits of animals, conspire to prove that while there is similarity in the general plan upon which they were created or formed, there is too great permanency of type to justify the idea that a transmutation of animals could ever have taken place at any prehistoric period of time. The differentiation of animals of the same type, race or genus was as clearly manifest four thousand years ago, as it is to-day, and certainly no transmutation has taken place within fifty centuries at least. The introduction of

*new* law at the present time, or at any other time since the harmonious arrangement was completed for the government of animal existence, would at once so disturb the equilibrium of existing laws as to ultimately destroy the prevailing order of things in the physical, and probably also in the moral world! Would not such an introduction or intrusion have been productive of similar results at any previous period since the finished plan of creation? If existing law prohibits the frequent breeding *inter se*, of near blood relatives, now, under penalty of physical and mental degeneration and ultimate extinction of the offspring of such family or families: Has not such law continued commensurate with the existence of such a family? And if true of one, why not equally so of any other type, race or family of mankind?

It was necessary, therefore, that Cain should take, as a companion, a woman in the land of his refuge, and the other sons of Adam find their wives among the *fair* daughters of men, as the Bible expressly declares that they did. We hold that the inter-marriage of the sons of Adam with another race, or races, was a physiological necessity for the propagation and *perpetuation* of the Adamite or Jewish race! and thus is demonstrated conclusively, as far as human reason and experience can prove anything, the *physiological impossibility* of any of the human races having descended from a few, much less a single pair in their beginning! No doubt nor uncertainty should remain (where the facts are known) on the mind of any one capable of defining and appreciating race distinctions, that the Adamite family was the *last* created type of the existing human races! and that it was intended for a purpose so grand and important to all the varieties of men, that its origin and history, should be so well established that no cloud, nor even a shadow, might obscure the medium through which the manifestation of "God in the flesh" was to be known in the great plan of human redemption. This glorious achievement, the result of almighty Wisdom and Power, was not designed for one, but for *all the races* which the "Lord God had created and made." St. Paul speaks very positively and authoritatively on this point, when he declares: "For until the law sin was in the world; but sin is not imputed when there is no law." "Nevertheless, death reigned from Adam to Moses, *even over them* that had not sinned after the similitude of Adam's transgression, *who is the figure of him* that was to come." (Rom. 5, 13 and 14.) The Old Bible, which is mainly an epitomized history of the Adamites, or Jews, from the time of their first appearance upon the earth to their establishment in Palestine, after the Noachian deluge, that swept away and destroyed so many of their race; contains certain statements and allusions to contemporaneous types or tribes of men in no wise related by blood, or race, to the Adamite branch of the Caucasian family. The Jewish race presents a remarkable and unmistakable evidence of *permanency* of type. The iconisms of this race, found in the temples of Egypt, date certainly as far back as the days of Abraham, and yet they would serve as correct portraits of the Jew of the present time! And all know, who have given any attention to ethnography at all, that even an intelligent child would be able to distinguish the unmixed Jew when once made familiar with his facial traits and expression of countenance, in any city on the globe, and from any and all other peoples and nationalities with which he might be mingled, and that too at a single glance!

The following statements and quotations from the Bible will clearly show that there was at least one other distinct race, contemporaneous with the Adamite family, or Jewish race, from the very dawn of its origin, if indeed it was not of a greatly anterior date. In Genesis, (our King James' vision of the Bible) the words *Adam* and *ish*, in the original Hebrew, are loosely and indiscriminately used by the translators, as though they were the same word. They have both been translated *man*, when in fact, the latter (*ish*) is used generically in the original and not in the particular sense of the former word. In the Hebrew text, "God (Elohim) said let us make *Adam* in our image," instead of "Make *man* in our image," as rendered in Gen. 1, 26. In the succeeding verse the Hebrew is: "So (Elohim) God created *the Adam*, or the *Adamite*" (ha-Adam): and the King James' Bible renders it: "So God created man in his own image," etc. It will be perceived that the Hebrew has the definite article pre-fixed, and the passage reads: So Elohim created "*the Adam*," or "*the Adamite*," (ha-Adam). In the second chapter, also, it was "*the Adam*," or "*the Adamite*," that the Creator formed from the dust of the earth, and who it was that disobeyed and fell, who was the husband of Eve, and the father of Abel and others. Hebrew scholars inform us that the expression *ha-Adam* does not designate the individual subsequent to the birth of Cain, as he was not "*the Adamite*" after the advent of his children, as then there were others of his own kind and likeness.

He is ever after, until his death, called only Adam. The expression *ha-Adam* is not used again until after his death, when it occurs to designate or specify *his descendents.* "And the Lord said, My Spirit shall not alway strive with *the Adamite*, for that *he* also is flesh," etc. "And it repented the Lord that he had made *the Adamite* on the earth;" and again, "the Lord said, I will destroy *the Adamite*," etc. The Hebrew text uses the word (*ish*) and not *the Adamite* when speaking of mankind generally; and when speaking of the relationship of husband and wife, as of Adam and Eve, the word for woman is *ishah*, the feminine of *ish*. Again Eve uses the word *ish* at the birth of Cain. "I have gotten a man, (ish) a male, "from the Lord." In many subsequent passages the terms "*Adam*" and "*ish*" are used in a manner that clearly shows the distinction existing between them; the latter referring to the lower, and the former to the higher, or Adamite race, as the Hebrew historian always desired to place the Jewish people, or Adamites. "Give ear, all ye inhabitants of the world, both *high* and *low*." Psalms 49, 1 and 2. Experts in the Hebrew inform us that the literal meaning of these words is "*sons of Adam*" and "*sons of man*" (ish). Again, in Psalms 42, 9, "Surely *men of low degree* (sons of *ish*), are vanity, and *men of high degree* (sons of *ha-Adam*) are a lie." Similar evidence of the recognition of races, other than the Adamites, may be found, Is. 2, 9; 5, 15; 31, 8; and Ezek. 23, 42. Other testimony on this interesting point might be cited from the Bible, but the foregoing must suffice for the present.

Professor Winchell says, in his admirable work noticed in the November issue of this journal: "From the date of the *earliest* records the Jew has been a *recognizable Jew*, the *negro* has been *distinctly a negro*, and the Egyptian and the Aryan and the Abyssinian have stood forth as *completely* differentiated as *they* appear to be *at present.* (*Preadamite*, p. 187.) And M. Charnay gives us some conclusive evidence in his letter of April 30, 1880, dated at *Vera Cruz*, and published in the *North American Review* for September, ultimo, on both the *diversity* or *plurality* of the races, and the *permanency* of the *distinctive* characteristics of the *three* primordial races, referred to already in this paper! In speaking of the remains of the ancient and famous Teotihuacan, or "City of the Gods," in Mexico, where he "found some one hundred and twenty-five heads of idols or *lares* and divers other objects." "One of these specimens is a *negro's head*

with ***thick lips*** and ***woolly hair***, ***all*** perfectly designed; the other, ***the face of a woman***, rather disfigured by a broken nose, but plainly of ***European*** or ***Grecian type***, and reminding us, by its features, of the Venus of Milo!" "This looks like a pleasantry; but no—my *photographs will prove that I simply state facts.*" "It must not be forgotten that we are here in the land of mysteries as regards *history and race.*"

In his fourth communication to the *Review*, received since the foregoing portion of this article was written, M. Charnay quotes from his diary of August 24th, 1880, made at Tula, in Mexico, the supposed ancient metropolis of the Toltecs, now, however, only a village of fifteen hundred inhabitants, situated about sixty-five miles to the north of the City of Mexico, where he also made extensive excavations.

He says: "In writing of Teotihuacean, I mentioned my having found, among certain little masks of terra-cotta, figures of negroes, etc. I also spoke of a figure resembling the Venus of Milo; and *now* I find at Tula a ***mold***, of which I have made a cast in stearine, which shows ***a European figure***, with the ***hair*** arranged in ***modern style***, and ***crowned*** with a ***braid of false hair!*** " I need hardly say these statements are marvellous, indeed, and but for the source from whence they emanate would be regarded as incredible.

Who were the builders of Uxmal, of Palenque, of Copan, of Milla, and of Chichen-itza? Doubtless they were a more highly civilized and cultured people even than those who built the "City of the Gods!" Those who designed and created these grand and stupendous structures, which even the defacing and destroying hand of time has not yet been able to level into dust, existed long ages if not eons ere Egypt was young or Greece had yet been born! Sufficient is known to satisfy a reasonable mind that the head of the Adamite, or Jewish race was of comparatively late appearance on the earth, and was unquestionably the *last* created type of the genus homo. We venture to assert, and place upon permanent record, that the Bible and science, when carefully compared with reason and common sense, will clearly show that the human race had not one only, but several, if not many distinct and separate foci of creation, and that there never has been, and never can be a successful and permanent transmutation of *any* of the prominent types of mankind! The Jew has existed in all latitudes and climes unchanged in his typical and ethnological characteristics, and though he has been cotemporary, and mingled with other types of the Caucasian family, he has neither lost, nor imparted to those among whom he has lived and moved, any of his physiognomic traits and characteristics, save in rare instances, where he has inter-married with other varieties of the white race! And in these cases the *facial* traits of the Jew, will be marked more or less, in his descendants for generations, even where they continue to intermarry with other branches of the Caucasian family! During the unnumbered centuries he has lived among others of the white race, he has remained like the wonderful gulf-stream, as it flows through the great ocean, in the midst of it, yet mingling not with its waters. And as whether in calm, or when convulsed and tempest-tossed, the water of the stream remains the same, so the Jewish stream flows on amid peace or war or famine or plenty, the same, separate, distinct and peculiar, yet sublime, amid the other races of mankind. Such is now the law! Was it not always so? On a future occasion attention will be invited to human *hybridity*, as further evidence of the diverse, or plural origin of the human races, and the importance of the subject to the medical practitioner will be shown, as far as the brief limits of a journal article will allow.

225 North Gilmor Street, December, 1880.

